# Optimising recruitment in clinical trials for progressive multiple sclerosis: observational analysis from the MS-SMART and MS-STAT2 randomised controlled trials

**DOI:** 10.1186/s13063-022-06588-z

**Published:** 2022-08-09

**Authors:** Thomas Williams, Sarah Alexander, James Blackstone, Floriana De Angelis, Nevin John, Anisha Doshi, Judy Beveridge, Marie Braisher, Emma Gray, Jeremy Chataway, on behalf of the MS-SMART and MS-STAT2 Investigators

**Affiliations:** 1 Queen Square Multiple Sclerosis Centre, Department of Neuroinflammation, UCL Queen Square Institute of Neurology, Faculty of Brain Sciences, University College London, London, WC1B 5EH UK; 2 Comprehensive Clinical Trials Unit, Institute of Clinical Trials and Methodology, University College London, London, UK; 3 MS-STAT2 Trial Patient and Public Involvement Committee, London, UK; 4 Multiple Sclerosis Society (UK), Carriage House, 8 City North Place, London, UK; 5 National Institute for Health Research, University College London Hospitals, Biomedical Research Centre, London, UK; 6 Medical Research Council Clinical Trials Unit at UCL, Institute of Clinical Trials and Methodology, University College London, London, UK

**Keywords:** Clinical trials, Recruitment, Multiple sclerosis

## Abstract

**Background:**

Slower than planned recruitment is a major factor contributing to the delay or failure of randomised controlled trials to report on time. There is a limited evidence base regarding the optimisation of recruitment strategies. Here we performed an observational review of our experience in recruitment for two large randomised controlled trials for people with secondary progressive multiple sclerosis. We aimed to explicitly determine those factors which can facilitate trial recruitment in progressive neurodegenerative disease.

**Methods:**

Recruitment data from the sequential MS-SMART [NCT01910259] and MS-STAT2 [NCT03387670] UK randomised controlled trials was reviewed from the largest recruiting site, University College London (UCL). The trial population was similar which allowed comparison over the two recruitment periods of 2015–2016 and 2018–2021. This included sources of referral, progress through stages of recruitment, reasons for participant ineligibility and the impact of publicity events upon recruitment.

**Results:**

In MS-SMART, 18% of patients contacted were enrolled, compared to 27% for MS-STAT2. Online registration of interest portals provided the greatest number of referrals (76% in MS-SMART, and 51% in MS-STAT2), with publicity in national media outlets producing a demonstrable increase in the number of potential participants. The introduction of an online self-screening questionnaire for MS-STAT2 resulted in 67% of potential participants (3080 of 4605) automatically determining their own *ineligibility*. In both studies, however, around 60% of those directly telephoned to discuss the study were not eligible, with difficulties related to travel to trial visits, or excluded medication, being the most common issues. Eighty-four percent of those deemed potentially eligible following telephone calls were enrolled in the MS-STAT2 study, compared to only 55% for MS-SMART.

**Conclusions:**

Through a detailed review of recruiting participants at the largest centre into two large randomised controlled trials with similar entry criteria, we have identified a number of approaches that may improve recruitment efficiency. We highlight here the importance of mandatory online self-screening questionnaires, a coordinated publicity campaign, and simple interventions such as eligibility checklists and appointment reminders. Recruitment approaches should be further assessed through a studies within a trial (SWAT) design.

**Trial registration:**

MS-SMART: NCT01910259; registered July 2013 and MS-STAT2: NCT03387670; registered Jan 2018

**Supplementary Information:**

The online version contains supplementary material available at 10.1186/s13063-022-06588-z.

## Background

Randomised controlled trials (RCTs) are the gold-standard for assessing the efficacy and safety of medical treatments [1, 2]. Speed of recruitment is one vital parameter which influences trial cost and time to clinical practice. Slow recruitment is the most commonly cited reason for delayed trial results, frequently necessitating additional resources, trial extensions or contributing to trial failure [3–5]. A recent systematic review has highlighted the paucity of evidence upon which recruitment strategies are based, and identifying successful strategies to improve the efficiency of trial recruitment has been stated as a major research priority [6–8]. Such issues are prevalent in trials for progressive neurological conditions, such as multiple sclerosis, where our ability to develop new treatments can be limited by inefficiencies in the operational aspects of clinical trial execution. Here we describe our detailed experience from two large, academically led, non-commercial UK multi-centre RCTs in secondary progressive multiple sclerosis (SPMS) with the objective of identifying methods to optimise the efficiency of clinical trial recruitment for people with progressive neurological conditions.

## Methods

### The MS-SMART and MS-STAT2 clinical trials

MS-SMART (NCT01910259) was a multi-centre phase 2b RCT simultaneously assessing the efficacy of 3 potentially neuroprotective treatments in slowing the rate of brain atrophy compared to placebo in participants with SPMS [9]. It involved 13 sites across the UK, and enrolled 445 participants. Recruitment began in February 2015 and finished in May 2016. University College London (UCL) was the largest trial site, recruiting 40% of the trial cohort.

MS-STAT2 (NCT03387670) is an ongoing multi-centre phase 3 RCT assessing the efficacy of high-dose simvastatin compared to placebo in slowing the rate of disability progression in SPMS. It involves 31 trial sites across the UK, recruiting 964 participants. Recruitment began in May 2018 and finished in September 2021. UCL was again the largest trial site, recruiting 33% of the trial cohort.

In both MS-SMART and MS-STAT2 trials, detailed recruitment data was systemically recorded at the main UCL site. Our analysis of recruitment data is therefore restricted to the UCL site alone, which contributed around a third of the total cohorts.

The trial populations recruited at the UCL site were similar in MS-SMART and MS-STAT2, as shown in Table 1. Patients had established SPMS, with median age of 55 years, and median EDSS of 6.0. Full eligibility criteria are available from references [9, 10].

**Table 1:** Participants, intervention, comparison and outcome for the UCL MS-SMART and UCL MS-STAT2 clinical trial cohorts

	UCL MS-SMART	UCL MS-STAT2
Participants:	176 people with SPMS	315 people with SPMS
Age (years)	55 (34 to 65)	55 (32 to 65)
EDSS	6.0 (4.0 to 6.5)	6.0 (4.0 to 6.5)
Intervention	Fluoxetine, Riluzole or Amiloride	Simvastatin
Comparison (over encapsulated)	Placebo	Placebo
Outcome	Percentage whole brain volume change	Time to confirmed disability progression on EDSS

Characteristics of the MS-SMART and MS-STAT2 trial populations at the UCL site included in this analysis. Age and EDSS are presented as median (range)

*UCL* University College London, *SPMS* secondary progressive multiple sclerosis, EDSS expanded disability status scale

### Methods of recruitment

In both MS-SMART and MS-STAT2, recruitment followed a three-step process. Firstly, referrals were collected via multiple sources, including online registration of interest portals (hereafter referred to as online portals), local databases, neurology consultant or other clinician referrals, patient self-referrals and patient outreach meetings at local MS centres. Secondly, appropriate referrals were then manually contacted for telephone *pre-screening* to discuss trial participation and review eligibility criteria. This telephone pre-screening was performed solely by clinical research fellows with experience in caring for people with MS and research trials. It was performed ad hoc in MS-SMART, and this experience prompted the development of formalised pre-screening eligibility checklists, which were used throughout MS-STAT2 (see supplementary material). Finally, patients passing telephone pre-screening were then invited to a face-to-face screening appointment where eligibility was further assessed prior to enrolment. Reminder text messages were introduced for these appointments in MS-STAT2 due to the high rates of non-attendance observed in MS-SMART.

### Registration of Interest Portals

The MS-SMART online portal was created on a web-based portal linked to a database maintained by the Edinburgh Clinical Trials Unit. Potential participants were directed to it via publicity generated by the UK Multiple Sclerosis Society, including national publicity campaigns. It collected name, contact details and a location to ascertain the most appropriate MS-SMART site to refer the potential participant to.

The MS-STAT2 online portal was created on the Research Electronic Data Capture (REDCap) platform using UK-MS Register infrastructure [11]. In addition to data on consent, patient location and contact details, a self-screening questionnaire was added for the MS-STAT2 online portal to determine if potential participants were likely to meet the trial eligibility criteria. This was designed with input from trial clinicians and a draft was reviewed by people with progressive MS who were members of the UK MS Society’s Research Network. Patients’ suggestions were incorporated into the final version of the questionnaire. Potential participants were directed to the online portal from the MS-STAT2 UCL webpage [12], as well as various MS charity websites, MS clinics and publicity events. If the online portal questionnaire suggested potential participants were unlikely to be eligible for the trial, they were automatically informed of this and not contacted.

### MS-STAT2 publicity events

Dates of publicity events for the MS-STAT2 trial were prospectively collected during recruitment, including UK Multiple Sclerosis Society Website updates and local and national media coverage. Additional publicity events were retrospectively identified through online searches.

### Analysis of recruitment

For the lead UCL site only, the main sources of recruitment and progress of participants through the stages of recruitment were compared between MS-SMART and MS-STAT2. Percentages obtained from differences sources and percentages passing each stage of recruitment were calculated from research databases, identifying the major cause of ineligibility at each stage. In the rare event of participants being referred twice from different sources, only the first referral source was included.

The impact of various publicity events on trial recruitment is assessed for MS-STAT2 only, by qualitatively comparing UK-wide activity on the online portal to the timing of various publicity events. A 7-day rolling average of completed online portal entries was calculated and then inspected for temporal associations with recent publicity events.

## Results

### Sources of referral

**Fig. 1:**
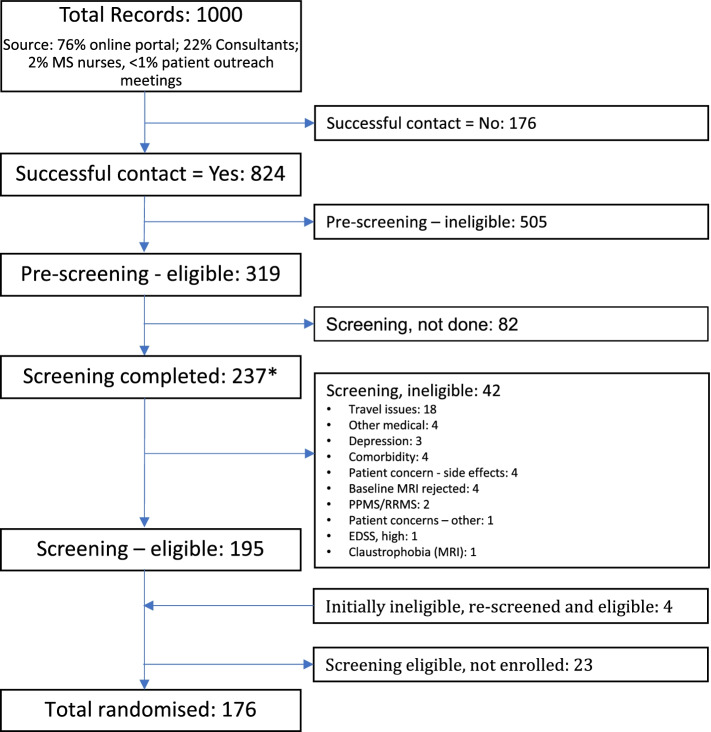
Participant recruitment and reasons for ineligibility at the lead UCL site for MS-SMART. *As this data is derived from the lead UCL MS-SMART site only, this number (237 participants attending screening) differs from the total number of participants screened across all UK sites (547), which is reported in reference [9]. RRMS, relapsing remitting multiple sclerosis; PPMS, primary progressive multiple sclerosis; EDSS, Expanded Disability Status Scale

**Fig. 2:**
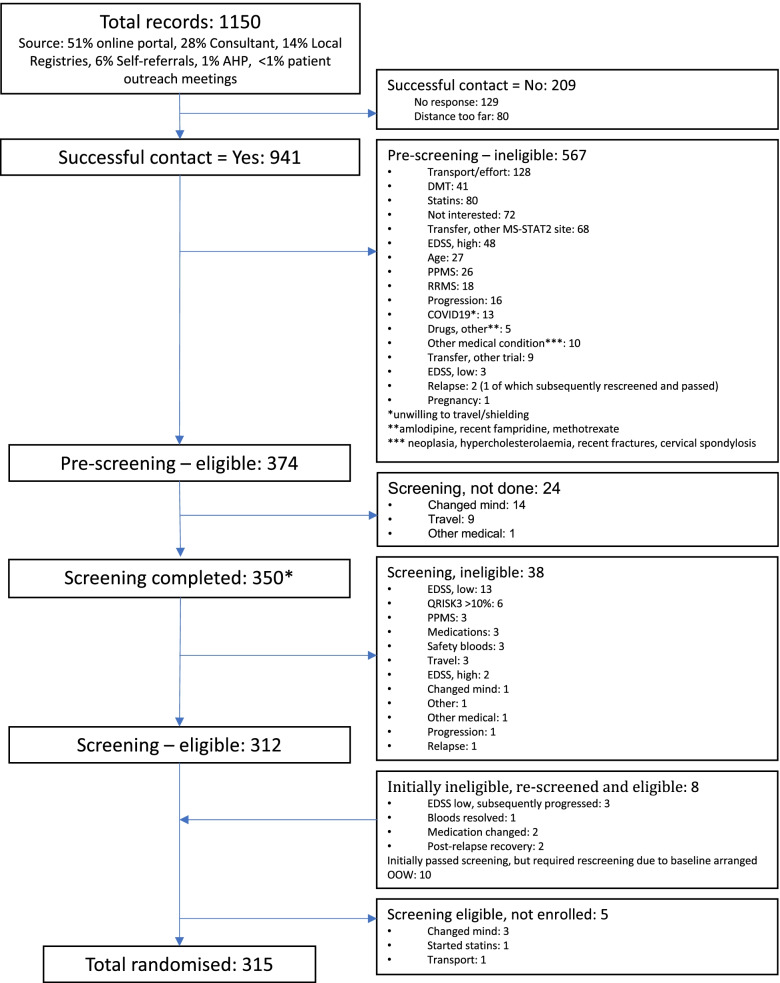
Participant recruitment and reasons for ineligibility at the lead UCL site for MS-STAT2. *As for Fig. 1, as this data is derived from the lead UCL MS-STAT2 site only, the number screened here will be lower that for the whole trial cohort. AHP, allied health professional; DMT, disease-modifying therapy; RRMS, relapsing remitting multiple sclerosis; PPMS, primary progressive multiple sclerosis; EDSS, Expanded Disability Status Scale; QRISK3, cardiovascular risk calculator [13]; OOW, Out of Window (for trial scheduled visit)

Sources of referral for MS-SMART (Fig. 1) and MS-STAT2 (Fig. 2) to the UCL trial site were similar . In MS-SMART, 76% of referrals were obtained from the online portal, 22% from consultant referrals, 2% from MS nurses and <1% from patient outreach meetings. In MS-STAT2, 51% of referrals were obtained from the online portal, 28% from consultant referrals, 14% from local research registries, 6% self-referrals, 1% from other allied health professionals and <1% from visits to patient outreach meetings (rounded figures).

Data on the number of participants randomised by each recruitment source were known for MS-STAT2 only. Whilst the online portal generated the greatest number of referrals (51%), a smaller percentage of such patients reached randomisation compared to consultant referrals (28%): 22% were randomised from the online portal compared to 35% from consultant referrals.

### Recruitment efficiency

Across all UK trial sites, the introduction of a self-screening questionnaire into the online portal for MS-STAT2 facilitated the early identification of many potential participants who were unlikely to be eligible for the trial. From a total of 4605 online portal entries, 3080 (67%) were automatically informed that they were unlikely to be eligible. The main criteria that excluded such candidates were the age limit (25–65 inclusive) and questions related to their ability to travel to trial sites.

The progress of potential participants through each stage of screening in the two trials is compared in Table 2. Overall recruitment efficiency (randomised patients as a percentage of all those manually considered) was 18% in MS-SMART and 27% in MS-STAT2.

**Table 2:** Comparison of recruitment efficiency between MS-SMART and MS-STAT2

	MS-SMART	MS-STAT2
Eligible at online portal self-screening questionnaire (UK-wide)	NA	1525/4605 (33%)
Successful contact^a^	824/1000 (82%)	941/1150 (82%)
Eligible after telephone pre-screening^b^	319/824 (39%)	374/941 (40%)
Pre-screened patients who attended face-to-face screening (%)^c^	237/319 (75%)	350/374 (94%)
Eligible at face-to-face screening^d^	195/237 (82%)	312/350 (89%)
Total randomised as % of all potential participants manually considered	176/1000 (18%)	315/1150 (27%)
Total randomised as % of those passing pre-screening	176/319 (55%)	315/374 (84%)
Total randomised as % of those who attended screening^d^	176/237 (74%)	315/350 (90%)

Data on the pass rate for the online portal questionnaire for MS-STAT2 are for UK-wide responses; all subsequent data are for the UCL sites only. *NA*, not applicable (as the MS-SMART online portal did not include a self-screening questionnaire)

^a^Those successfully contacted as a percentage of all potential participants manually considered

^b^Those eligible at telephone pre-screening as a percentage of those successfully contacted

^c^Those who attended a face to face screening appointment, as a percentage of those deemed potentially eligible at telephone pre-screening and invited to attend screening

^d^Those eligible at screening as a percentage of those attending screening, and total randomised as a percentage of those attending screening, produce different figures due to some patients who are eligible at screening not proceeding to randomisation, and some initially ineligible patients being rescreened if the eligibility issue could be resolved

### Impact of publicity events upon recruitment

**Fig. 3:**
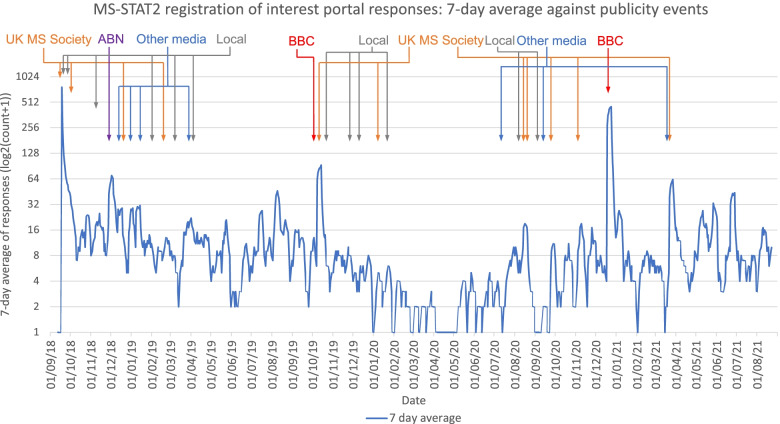
UK-wide potential participants completing the MS-STAT2 Registration of Interest Portal: temporal relationship with identifiable publicity events. The date and time that UK-wide potential participants completed the online portal for the MS-STAT2 study is used to create a 7-day rolling average of online portal responses. This is plotted against time from the launch of the online portal, with the timing of identifiable publicity events added so temporal relationships can be inferred. Note online portal responses are quantified on a log_2_(count + 1) scale for clarity. ABN, Association of British Neurologists; BBC, British Broadcasting Corporation

The temporal relationship between UK-wide MS-STAT2 online portal entry completions (shown as a 7-day average) and various publicity events carried out during the recruitment period are shown in Fig. 3.

As expected, the largest number of responses were generated at the opening of the online portal, which was coordinated with publicity via the UK Multiple Sclerosis Society. Subsequently, spikes in online portal completions appeared in association with national BBC publicity (October 2019 and December 2020) [14, 15].

It is harder to infer causality for smaller spikes in online portal completions. In December 2018, increasing interest was observed following coverage of MS-STAT2 in the Association of British Neurologists newsletter and website. This is sent by email to all neurologists in the UK, and it is plausible that this may have generated interest if neurologists subsequently mentioned it to their patients in the following weeks. In March 2021, an increase in interest coincided with the launch of the OCTOPUS trial (EudraCT: 2021-003034-37) (also for people with progressive MS). The UK Multiple Sclerosis Society and Guardian newspaper carried the story, and both referenced to the ongoing MS-STAT2 trial and contained the online portal link. Various smaller newspaper stories may have generated some additional interest. Overall, local events, such as the university website/newsletter coverage or visits to local MS centres, did not appear to generate a perceptible increase in online portal completions. The onset of the COVID-19 pandemic and the first UK national lockdown between March and June 2020 coincided with both reduced publicity events and reduced online portal completions.

### Comparison of overall recruitment for MS-SMART and MS-STAT2

**Fig. 4:**
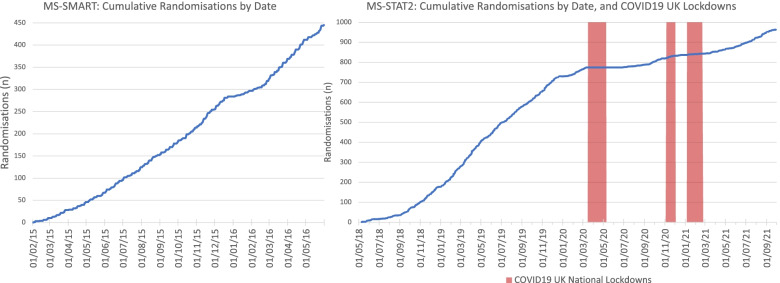
Overall UK-wide recruitment into MS-SMART and MS-STAT2. The overall randomisations in MS-SMART and MS-STAT2 are plotted against time from first randomisation in each study. For MS-STAT2, the timings of the UK National COVID19 Lockdowns are included, although milder / localised restrictions continued outside of these time periods

Figure 4 depicts the cumulative multi-site recruitment for MS-SMART and MS-STAT2, including dates of the UK COVID-19 national lockdowns for the latter. The pandemic had a clear impact upon the latter half of MS-STAT2 recruitment, the rates of which never recovered to those achieved in 2019.

## Discussion

We have reviewed here the operational aspects of clinical trial recruitment in progressive MS from two large academically led, non-commercial UK studies. This includes phase 2b and phase 3 experience at the major recruiting trial centre, with over 2000 intial contacts, and ultimately nearly 500 participants randomised. Based upon this experience, we summarise in Table 3 several key suggestions to improve the efficiency of recruitment, which are explored below. These may have practical implications for the management of future clinical trials, in this population, but also in neurodegenerative diseases in general.

**Table 3:** Optimising clinical trial recruitment: key points

Optimising clinical trial recruitment: key suggestions	
1. In the MS-SMART and MS-STAT2 trials for people with SPMS at UCL, between 4 and 6 potential participants were contacted for every 1 participant successfully recruited. Substantial resources are therefore required to meet recruitment targets. 2. For large, multi-centre trials, we find that it is most efficient to target efforts aimed at increasing participant referrals on national media outlets. Informing MS specialists about trials via professional organisations and regular supporting charity involvement also appeared beneficial. Attending smaller patient outreach meetings generates few referrals—though continuing these as remote teleconferences may improve efficiency 3. We find an online Registration of Interest Portal with accompanying eligibility questionnaire to be an essential source of high-volume referrals, although they require careful design and active management. We suggest that completion of such an online portal and questionnaire should be considered *mandatory* for all potential participants. The questionnaires may benefit from a greater emphasis on whether potential participants have considered the logistics and travel required to attend regular study visits 4. Telephone pre-screening is able to exclude the majority of ineligible participants. It is therefore essential in order to minimise face-to-face screening failures and may be improved through the use of detailed eligibility checklists. 5. We find that clear appointment instructions and text appointment reminders are simple interventions that may improve attendance at face-to-face screening appointments 6. Difficulty with travel to trial sites was the most common reason for failure to recruit potential participants. Efforts should be made to reduce the number of face-to-face visits and increasing travel expenses, where possible	

### Identification of potential participants

Our experience of trial publicity in MS-STAT2, as shown in Fig. 3, found that national events had a demonstrable impact upon the number of potential participants completing the MS-STAT2 online portal. This suggests that for large trials, publicity efforts should be focussed on achieving coverage in *national* media outlets. Continuing support from the UK Multiple Sclerosis Society, who provided regular updates on the trial via their website, in addition to coordinating larger media events, was essential for this. Weaker signals were obtained for smaller publicity events, but involvement of the Association of British Neurologists, aiming to increase neurologists’ awareness of the trial, is likely to have been additionally helpful. This may be particularly beneficial given the observation that consultant referrals to the MS-STAT2 trial were more likely to be randomised than potential participants obtained via other sources (35% of consultant referrals were randomised, compared to 22% from the online portal). We speculate that this difference is likely to relate to the consultants being skilled in determining potential trial eligibility, and with their consultants’ support, patients may be more enthusiastic about participating.

Smaller publicity events, such as visits to MS therapy centres, local presentations and updates to departmental websites, *do not appear* to have a quantifiable impact upon online portal completions. Certainly, the efforts involved in creating such events appear to be out of proportion to the recruitment outcomes that we are able to detect here. We acknowledge, however, that we cannot quantify the good will and any subsequent word of mouth publicity that may be derived from such events. Continuing such events as remote teleconferences, with the added efficiency that this can bring, may be a very useful option for the future.

### Assessing eligibility for potential participants

Both studies relied heavily on the online portals to generate and manage referrals, representing 76% of referrals to UCL in MS-SMART, and 51% of referrals to UCL in MS-STAT2. Combined with appropriate trial publicity, an online portal therefore appears to be an important aspect for recruitment in multi-centre trials in progressive MS.

Incorporating a self-screening questionnaire into an online portal is recognised as an effective strategy to facilitate quicker and more efficient identification of potentially eligible participants [16]. This is reflected in our experience, as shown by the 3080 potential participants (67%) who were automatically informed of their likely ineligibility following completion of the MS-STAT2 online portal self-screening questionnaire. Contacting 3000 ineligible patients would have been an enormous use of precious resource. Empowering candidates to quickly determine their own eligibility also importantly avoids the inevitable dissatisfaction of ineligible patients waiting many weeks to be contacted by a member of the trial team.

Despite the introduction of this important self-screening questionnaire for the MS-STAT2 trial, however, only 40% of potential participants were deemed eligible after the manual pre-screening telephone call, similar to the results found in MS-SMART (39%). With each pre-screening discussion typically taking up to 30 min, across the two trials, we estimate over 500 hs of specialist clinician time was spend discussing the studies with ultimately ineligible candidates.

In assessing why the rates of ineligibility remained similar between MS-SMART and MS-STAT2, despite the introduction of the self-screening questionnaire in MS-STAT2, it should firstly be noted that 49% of referrals for MS-STAT2 came from sources other than the online portal, and hence, these potential participants did not complete the self-screening questionnaire. Secondly, for those MS-STAT2 candidates that did complete the questionnaire and were deemed potentially *eligible*, the most common reasons for subsequent *ineligibility* at manual telephone pre-screening were as follows: transport/distance to visits (*n*=126), medication (*n*=98) and no longer interested in the study (*n*=25). Issues related to the specifics of their multiple sclerosis (type of MS, relapses, estimated EDSS and progression; total *n*=48) were a relatively less frequent reason for ineligibility at manual pre-screening after the self-screening questionnaire.

Once deemed eligible at pre-screening, 55% of participants reached randomisation in MS-SMART, compared to 84% in MS-STAT2. Whilst we cannot exclude a role of additional unidentified factors in contributing to this, we suggest that two simple interventions are likely to have contributed. Firstly, the introduction of detailed pre-screening checklists for MS-STAT2 may have contributed to the higher rate of eligibility at face to face screening. Secondly, the use of appointment reminders for MS-STAT2 is likely to have contributed to the improved attendance at screening visits (75% in MS-SMART, 94% in MS-STAT2).

For both trials, the most common reason for either pre-screening or screening ineligibility related to issues with transport to trial visits (responsible for 43% of screening failure in MS-SMART, and 22% of all ineligible patients in MS-STAT2), followed by contraindicated medication (disease-modifying therapy, antidepressants (MS-SMART) or statins (MS-STAT2)) (21% of all eligible patients in MS-STAT2).

Overall, we conclude from our experience of assessing eligibility that firstly, all potential participants, regardless of their referral source, should be directed to a mandatory trial online portal, including a self-screening questionnaire. This should improve efficiency both for trial staff and participants by ensuring that all candidates have the opportunity to automatically determine their own likely eligibility. The resources required to establish such online portals should clearly be considered as part of trial funding applications.

Additionally, emphasis should be placed on identifying and overcoming logistical barriers to trial participation, as these are the most common limitations to recruitment. When designing the online portal questionnaires, clear details on the locations of participating trial sites should be included, and such sites should be geographically well distributed to improve inclusion. The number of appointments requiring face to face attendance should be detailed, together with increasing use of remote visits where possible (see below). With this information, potential participants should then be asked to indicate, as part of self-screening, whether they feel able to meet the burden of visits required by the trial, and whether they have considered the logistics of regular travel to the trial sites. If candidates feel unable to meet such requirements of a trial, they should be provided with a mechanism to report the reason for this, allowing trial sites to troubleshoot the issues and explore opportunities to overcome them, aiming to maximise inclusion.

Further improvements in identifying medical exclusions, particularly related to the common finding of contra-indicated medications, may additionally be achieved by allowing potential participants access to update their submitted details (for example, when their medications change).

### Overcoming issues that contribute to ineligibility

There is increasing interest in the remote assessment of participants in clinical trials, the importance of which was additionally highlighted due to the impact of the COVID-19 pandemic on clinical trials [17, 18]. As shown in Fig. 4, MS-STAT2 recruitment was severely impacted by COVID-19, when legal restrictions and safety concerns limited participants’ ability to travel. Validated remote forms for many of the assessments required for MS-SMART and MS-STAT2 enrolment are available, though concerns persist around the reliability of remotely collected data; the ability of participants, particularly those with cognitive impairment, to provide informed consent remotely; and limitations that particularly apply when MRI acquisition is an obligatory eligibility requirement [19–21]. Our current view is that remote assessments should increasingly be used to replace some follow-up assessments, thereby reducing travel requirements, but with the initial enrolment and important end-points still obtained face to face, where possible. Caution, however, should be taken when increasing the use of technology within recruitment (online self-screening questionnaires) and remote assessments (tele-conferencing or online questionnaires for data collection) in order to ensure such interfaces are accessible to the target patient population; enhanced PPI involvement to rigorously assess such mechanisms will be essential [22].

### Existing literature on trial recruitment strategies

Within the MS literature, a previous analysis from an exercise-based RCT (ExIMS) found that the majority (60%) of participants were recruited from MS hospital clinics [23]. This approach was, however, costly in terms of the time required for researchers to attend each clinic (4.2 h per participant recruited, compared to 0.6 h for those sent mailed invites) [23]. In contrast to the MS-SMART and MS-STAT2 trials, the ExIMS trial did not use media publicity due to concerns that it may prompt contact from many ineligible participants. We suggest that such concerns may be overcome by combining media campaigns with subsequent self-screening questionnaires.

Beyond MS, a Cochrane review of randomised studies investigating trial recruitment found limited evidence to support recruitment approaches [6]. Open as opposed to blinded trial designs appear to improve recruitment, but will compromise the quality of evidence generated [6]. Similar to our conclusions, telephone reminders have previously been shown to increase recruitment for patients unresponsive to postal invitations [6]. Highlighting the limited number of available trial places, or including quotes from previous participants, may also be beneficial [6].

More recently, programmes such as PROMETHEUS (Promoting the use of Studies within a Trial) have facilitated the generation of higher quality evidence regarding recruitment processes [24]. These have made clear that adaptations to patient information leaflets or inclusion of multi-media information resources, for example, appear to have little effect upon recruitment [25]. Personalised text message prompts, as opposed to the generic reminders we introduced for MS-STAT2, however, may increase response rates [26].

Non-randomised studies reporting on recruitment strategies provide a more limited evidence base [27]. Our experience regarding the value of including the Association of British Neurologists network in trial publicity is, however, supported by previous reports highlighting the benefits of disseminating information about RCTs via professional organisations [28]. For future trials, increasing use of social media to disseminate such information may be an additionally useful strategy [29]. The communication skills of the trial staff involved in recruitment may also be important to efficient recruitment, and training of such staff may improve confidence [30, 31]. Our experience regarding the benefits of incorporating of a self-screening questionnaire into online trial registration portals also matches that reported by others [16].

### Limitations

As highlighted above, the main limitation of this study is the observational design. We therefore cannot demonstrate causality for the inferences we have made through comparison of our experience of trial recruitment in MS-SMART and MS-STAT2. Other, unidentified factors may also have contributed to differences in recruitment between the two studies. Building upon our observations, future research should therefore focus on improving the evidence base for recruitment strategies through prospective, randomised *studies within a trial* (SWATs) [32]. These should ideally be coordinated through organisations such as the Trials Forge SWAT Network to establish research priorities and minimise research waste [33].

## Conclusions

We have described our experience of recruiting participants with SPMS into two large RCTs in order to identify areas where the efficiency of recruitment could be improved. We have outlined in Table 3 a number of key areas which we suggest may be helpful in improving the efficiency of recruitment into future clinical trials, and in particular those for neurodegenerative diseases. Future research should aim to build upon our observations, ideally through prospective, randomised SWAT designs. Beyond these, further research with considerable PPI engagement is required in order to identify the most effective ways of reducing the barriers to recruitment from the patients’ perspective, aiming to achieve more equitable access to research from across a diverse patient population.

## Supplementary Information

**Additional file 1.** Prescreen Checklist. Investigator groups.

## Data Availability

The datasets generated and/or analysed during the current study are not publicly available due to the patient-identifiable material contained, but fully anonymised datasets are available from the corresponding author on reasonable request.

## Abbreviations

MRC: Medical Research Council
NIHR: National Institute for Health Research
UCLH: University College London Hospitals
HTA: Health Technology Assessment
UCL: University College London
RCTs: Randomised controlled trials
SPMS: Secondary progressive multiple sclerosis
REDCap: Research Electronic Data Capture
PPI: Patient and public involvement
AHP: Allied health professional
DMT: Disease-modifying therapy
RRMS: Relapsing remitting multiple sclerosis
PPMS: Primary progressive multiple sclerosis
EDSS: Expanded Disability Status Scale
OOW: Out of Window (for trial scheduled visit)
NA: Not applicable
