# Supplementary material for: Optimising recruitment in clinical trials for progressive multiple sclerosis: observational analysis from the MS-SMART and MS-STAT2 randomised controlled trials
Source: Trials. 2022 Aug 9;23:644. doi: 10.1186/s13063-022-06588-z (PMC9361231; doi:10.1186/s13063-022-06588-z)
Supplement: Supplementary file 1 — Additional file 1. Prescreen Checklist. Investigator groups. [file 13063_2022_6588_MOESM1_ESM.zip › OptimisingRecruitmentPMS_supplement_InvestigatorGroupsR1.docx]

MS-SMART Investigators:

Jeremy Chataway, Claudia A M Gandini Wheeler-Kingshott, Floriana De Angelis, Domenico Plantone, Anisha Doshi, Nevin John, Thomas Williams, Jonathan Stutters, Ferran Prados Carrasco, David MacManus, Frederik Barkhof, Sebastien Ourselin, Marie Braisher, Tiggy Beyene, Vanessa Bassan, Alvin Zapata (Queen Square Multiple Sclerosis Centre, University College London and University College London Hospitals NHS Foundation Trust, London, UK); Siddharthan Chandran, Peter Connick, Dawn Lyle, James Cameron, Daisy Mollison, Shuna Colville, Baljean Dhillon (Anne Rowling Regenerative Neurology Clinic, The University of Edinburgh, Royal Infirmary of Edinburgh, NHS Lothian, Edinburgh, UK); Christopher J Weir, Richard A Parker, Moira Ross, Gina Cranswick, Allan Walker, Lorraine Smith (Edinburgh Clinical Trials Unit [ECTU], Usher Institute, University of Edinburgh, Edinburgh, UK); Gavin Giovannoni, Sharmilee Gnanapavan (Blizard Institute, Barts and The London School of Medicine and Dentistry, Queen Mary University, Barts Health NHS Trust, London, UK); Richard Nicholas (Imperial College Healthcare NHS Trust, London, UK); Waqar Rashid, Julia Aram (Brighton and Sussex University Hospitals NHS Trust, Brighton, UK); Helen Ford (Leeds General Infirmary, Leeds Teaching Hospitals NHS Trust, Leeds, UK); Sue H Pavitt (Dental Translational and Clinical Research Unit, University of Leeds, Leeds, UK); James Overell (The Queen Elizabeth University Hospital Glasgow, NHS Greater Glasgow and Clyde, Glasgow, UK); Carolyn Young, Heinke Arndt (The Walton Centre NHS Foundation Trust, Liverpool, UK); Martin Duddy, Joe Guadagno (Royal Victoria Infirmary, The Newcastle upon Tyne Hospital NHS Foundation Trust, Newcastle, UK); Nikolaos Evangelou (Queens Medical Centre, Nottingham University Hospital NHS Trust, Nottingham, UK); Matthew Craner, Jacqueline Palace (John Radcliffe Hospital, Oxford University Hospitals NHS Foundation Trust, Oxford, UK); Jeremy Hobart (Derriford Hospital, University Hospitals Plymouth NHS Trust, Plymouth, UK); Basil Sharrack, David Paling (Royal Hallamshire Hospital, Sheffield Teaching Hospitals NHS Foundation Trust, Sheffield, UK); Clive Hawkins, Seema Kalra (Royal Stoke University Hospital, University Hospitals of North Midlands NHS Trust, Stoke?on?Trent, UK); Brendan McLean (Royal Cornwall Hospitals NHS Trust, Truro, UK); Nigel Stallard (Statistics and Epidemiology, Division of Health Sciences, Warwick Medical School, University of Warwick, Coventry, UK); and Roger Bastow (patient representative).

MS-STAT2 Investigators:

Jeremy Chataway, Thomas Williams, Nevin John, Floriana De Angelis, Alberto Calvi, Alessia Bianchi, Sarah Wright, Madiha Shatila, Anisha Doshi, Wallace Brownlee, Claudia A M Gandini Wheeler-Kingshott, Frederik Barkhof, Olga Ciccarelli, Jonathan Stutters, Ferran Prados Carrasco, Antonio Ricciardi, Marios Yiannakas, David MacManus, Megan Wynne, Marie Braisher (Queen Square Multiple Sclerosis Centre, University College London and University College London Hospitals NHS Foundation Trust, London, UK);

James Blackstone, Leanne Hockey, Josephine Parker, Jennifer Flight (Comprehensive Clinical Trials Unit [CCTU], Institute of Clinical Trials and Methodology, University College London, London, UK);

Chris Frost, Jennifer Nicholas (Centre for Statistical Methodology, London School of Hygiene and Tropical Medicine, London, UK);

Stuart Nixon and Judy Beveridge (patient representatives);

Siddharthan Chandran, Peter Connick, Dawn Lyle (Anne Rowling Regenerative Neurology Clinic, The University of Edinburgh, Royal Infirmary of Edinburgh, NHS Lothian, Edinburgh, UK);

Ian Galea, Elisabeth Jarman (University Hospital Southampton NHS Foundation Trust, Southampton, UK);

Helen Ford, Linford Fernandes, Maruthi Vinjam (Leeds Teaching Hospitals NHS Trust, Leeds, UK);

Sue Pavitt (Dental Translational and Clinical Research Unit, University of Leeds, Leeds, UK);

Basil Sharrack, David Paling (Sheffield Teaching Hospitals NHS Foundation Trust, Sheffield, UK);

Abdullah Shehu, Tarunya Arun, Mohamed Belhag (University Hospitals Coventry & Warwickshire NHS Trust, Coventry, UK);

Owen Pearson, Gillian Ingram, Christopher Rickards (Swansea Bay University Health Board, Swansea, UK);

Gavin McDonnell, Stella Hughes (Belfast Health and Social Care Trust, Belfast, UK);

Cord Spilker (Bradford Teaching Hospitals Foundation Trust, Bradford, UK);

Leonora Fisniku, Julia Aram (Brighton and Sussex University Hospitals NHS Trust, Brighton, UK);

Claire Rice (North Bristol NHS Trust, Bristol, UK);

Stefano Pluchino, Luca Peruzzotti-Jametti (Cambridge University Hospitals NHS Foundation Trust, Cambridge, UK)

Sreedharan Harikrishnan, Nikki Guck (East Kent Hospitals University NHS Foundation Trust, Canterbury, UK);

Neil Robertson, Emma Tallantyre (University Hospital of Wales, Cardiff, UK);

Timothy Harrower (Royal Devon University Healthcare NHS Foundation Trust, Exeter, UK);

Paul Gallagher (NHS Greater Glasgow and Clyde, Glasgow, UK);

Fayyaz Ahmed (Hull University Teaching Hospitals NHS Trust, Hull, UK);

Carolyn Young, Heike Arndt (The Walton Centre NHS Foundation Trust, Liverpool, UK);

Eli Silber (Lewisham and Greenwich NHS Trust, London, UK);

Richard Nicholas (Imperial College Healthcare NHS Trust, London, UK);

Martin Duddy (Royal Victoria Infirmary, The Newcastle upon Tyne Hospital NHS Foundation Trust, Newcastle, UK);

Martin Lee (Norfolk and Norwich University Hospitals NHS Foundation Trust, Norwich, UK);

Nikos Evangelou, Christopher Allen (Nottingham University Hospital NHS Trust, Nottingham, UK);

Matthew Craner, Ruth Geraldes (Oxford University Hospitals NHS Foundation Trust, Oxford, UK);

Jeremy Hobart (University Hospitals Plymouth NHS Trust, Plymouth, UK);

Charles Hillier (University Hospitals Dorset NHS Foundation Trust, Poole, UK);

Suresh Chhetri (Lancashire Teaching Hospitals NHS Foundation Trust, Preston, UK);

Miriam Mattoscio, Abhijit Chaudhuri (Barking, Havering and Redbridge University Hospitals NHS Trust, Romford, UK);

Seema Kalra (University Hospitals of North Midlands NHS Trust, Stoke-on-Trent, UK);

Agne Straukiene (Torbay & South Devon NHS Foundation Trust, Torbay, UK).
